# Investigation of Sequential Growth Factor Delivery during Cuprizone Challenge in Mice Aimed to Enhance Oligodendrogliogenesis and Myelin Repair

**DOI:** 10.1371/journal.pone.0063415

**Published:** 2013-05-01

**Authors:** Jennifer K. Sabo, Tim D. Aumann, Trevor J. Kilpatrick, Holly S. Cate

**Affiliations:** 1 Centre for Neuroscience Research, Department of Anatomy and Neuroscience, University of Melbourne, Melbourne, Australia; 2 Florey Institute of Neuroscience and Mental Health, University of Melbourne, Melbourne, Australia; Université Pierre et Marie Curie-Paris6, INSERM, CNRS, France

## Abstract

Repair in multiple sclerosis involves remyelination, a process in which axons are provided with a new myelin sheath by new oligodendrocytes. Bone morphogenic proteins (BMPs) are a family of growth factors that have been shown to influence the response of oligodendrocyte progenitor cells (OPCs) *in vivo* during demyelination and remyelination in the adult brain. We have previously shown that BMP4 infusion increases numbers of OPCs during cuprizone-induced demyelination, while infusion of Noggin, an endogenenous antagonist of BMP4 increases numbers of mature oligodendrocytes and remyelinated axons following recovery. Additional studies have shown that insulin-like growth factor-1 (IGF-1) promotes the survival of OPCs during cuprizone-induced demyelination. Based on these data, we investigated whether myelin repair could be further enhanced by sequential infusion of these agents firstly, BMP4 to increase OPC numbers, followed by either Noggin or IGF-1 to increase the differentiation and survival of the newly generated OPCs. We identified that sequential delivery of BMP4 and IGF-1 during cuprizone challenge increased the number of mature oligodendrocytes and decreased astrocyte numbers following recovery compared with vehicle infused mice, but did not alter remyelination. However, sequential delivery of BMP4 and Noggin during cuprizone challenge did not alter numbers of oligodendrocytes or astrocytes in the corpus callosum compared with vehicle infused mice. Furthermore, electron microscopy analysis revealed no change in average myelin thickness in the corpus callosum between vehicle infused and BMP4-Noggin infused mice. Our results suggest that while single delivery of Noggin or IGF-1 increased the production of mature oligodendrocytes *in vivo* in the context of demyelination, only Noggin infusion promoted remyelination. Thus, sequential delivery of BMP4 and Noggin or IGF-1 does not further enhance myelin repair above what occurs with delivery of Noggin alone.

## Introduction

Survival of oligodendrocytes is crucial for myelin integrity, which allows rapid saltatory conduction of action potentials to occur along axons [1]. In demyelinating diseases such as Multiple Sclerosis, oligodendrocytes undergo apoptotic death [2], which can lead to axons losing their myelin sheaths, degeneration of the axon and neuronal loss [1]. A promising strategy for treatment in MS is enhancement of remyelination, a process that restores myelin to denuded axons through the generation of oligodendrocytes from endogenous oligodendrocyte progenitor cells (OPCs) [3]. Remyelination can be impaired due to defects in OPC recruitment and differentiation into remyelinating oligodendrocytes [3]–[5]. Growth factors aimed at promoting the survival and differentiation of OPCs provide an attractive therapeutic target for the treatment of MS.

Several factors have been implicated in modulating OPC differentiation within the context of demyelination. Overexpression of epidermal growth factor (EGF) *in vivo* enhanced oligodendrogliogenesis and remyelination in lysolecithin-demyelinated corpus callosum [6]. The deletion of brain-derived neurotrophic factor (BDNF) *in vivo*, increased numbers of OPCs during cuprizone-induced demyelination and decreased levels of myelin proteins during remyelination, suggesting impairment in OPC differentiation [7]. Furthermore, work in our laboratory has shown that intraventricular infusion of Noggin, an inhibitor of Bone Morphogenic Protein (BMP) signalling, increased the number of oligodendrocytes within the remyelinating corpus callosum [8].

Insulin-like growth factor-1 (IGF-1) is a potent oligodendrocyte survival factor *in vitro* [9], [10] as well as *in vivo*. Transgenic overexpression of IGF-1 increases brain size, myelin thickness and numbers of myelinated axons [11], [12]. Conversely, IGF-1 knockout mice have small brains, reduced white matter tract size and densities of myelinated axons [13]. During cuprizone-induced demyelination, transgenic overexpression of IGF-1 promotes oligodendrocyte survival and limits the extent of demyelination [14]. Moreover, genetic ablation of the type 1 IGF receptor *in vivo* inhibited OPC survival and remyelination [5]. These studies suggest an important role for IGF-1 in OPC survival particularly in the context of demyelination.

We have previously reported that intraventricular infusion of BMP4 increased numbers of OPCs during cuprizone-induced demyelination, while infusion of Noggin increased numbers of mature oligodendrocytes and enhanced remyelination [8]. Here we report our findings from experiments aimed at determining whether remyelination could be further enhanced by sequential delivery of BMP4, to increase the pool of OPCs, followed by either Noggin or IGF-1, to increase OPC differentiation and survival.

## Experimental Procedures

### Ethics Statement

All experiments used wildtype C57BL/6 mice, which were obtained from the Animal Resource Centre (Canning Vale, Western Australia). All animal experiments were conducted according to National Health and Medical Research Council guidelines and approved by the Florey Institute's Animal Ethics Committee (Animal Ethics Committee number: 07-095). Animals undergoing surgery for cannula and osmotic pump implantation were thoroughly monitored for appropriate anaesthesia and recovery.

### Induction of demyelination and remyelination

Cuprizone mediated demyelination was induced as previously described [15]. For remyelination studies, mice were returned to normal chow for 1-week following cuprizone challenge.

### Intraventricular infusion

**Figure 1:**
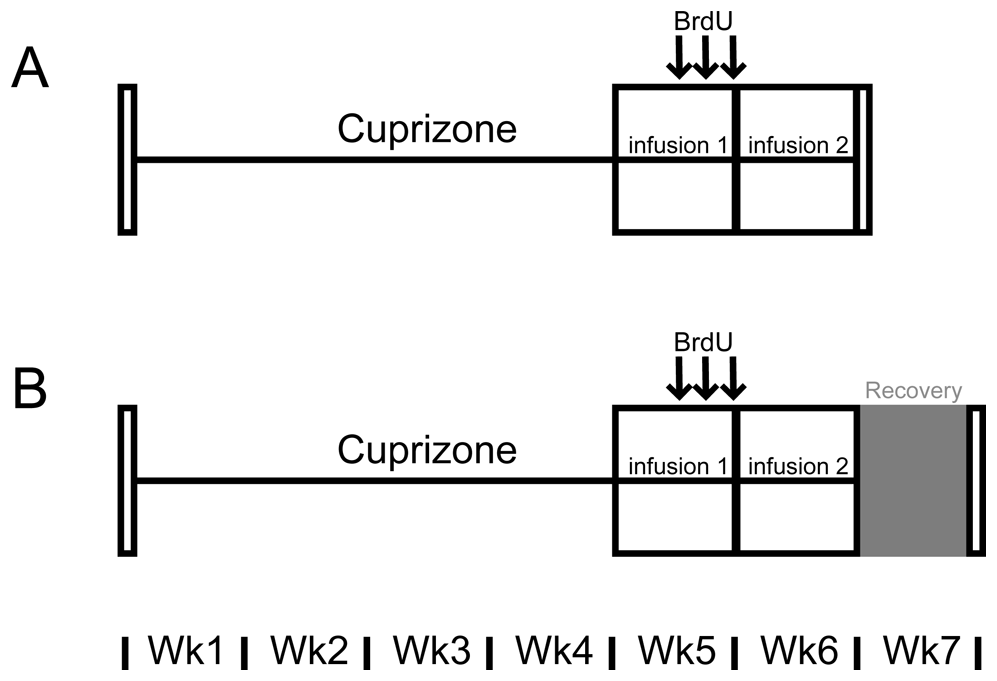
Timelines for infusion experiments. (A) For sequential delivery of growth factors during cuprizone challenge, mini-osmotic pumps were implanted after 4-weeks of cuprizone challenge to deliver either vehicle or BMP4 into the lateral ventricle for 7 days. Next, the mini-osmotic pump delivering vehicle or BMP4 was replaced with a new pump delivering vehicle, Noggin or IGF-1 for the final 7 days of a six-week cuprizone challenge. BrdU was added to the drinking water for the final 3 days of the first infusion. (B) The infusion paradigm was used as described above in A, however, the mice were allowed to recover for 1-week by removing cuprizone from their diet.

Recombinant human BMP4 or mouse Noggin (R&D Systems, Minneapolis, MN, USA) dissolved in artificial CSF (aCSF) at a dose of 400 ng per day or mouse insulin-like growth factor-1 (IGF-1) (Abcam) dissolved in aCSF at a dose of 1200 ng per day or aCSF was delivered into the lateral ventricle by mini-osmotic pumps (ALZET, Durect Corporation) (model 1002, 14 d infusion, 0.25 ml/h flow rate; model 1007, 5 and 7 d infusions, 0.50 ml/h flow rate) as previously described [15]. For sequential delivery, the ALZET pump delivering aCSF or BMP4 for 7 days was replaced with a new ALZET pump delivering Noggin or IGF-1 for 7 days. The pump being replaced was detached from the tube leading to the brain cannula, and the new pump was then attached to this tube. Mice received 1 mg/ml 5-bromodeoxyuridine (BrdU) (Sigma Aldrich, St Louis, MO, USA) in their drinking water for 3 d during the first infusion as indicated in the experimental timelines Figure 1.

### Histology and Immunohistochemistry

Mice were anesthetized and perfused intracardially with PBS followed by 4% paraformaldehyde. Brains were post-fixed for 30 minutes at 4°C and then equilibrated in 20% sucrose in PBS overnight at 4°C for cryoprotection before being embedded in Tissue-Tek O.C.T. (Sakura), frozen using isopentane (Ajax Finechem, Taren Point, New South Wales, Australia) on dry ice, and stored at −80°C. For fluorescence microscopy, 10 µm coronal cryostat sections were immunoprobed as previously described [15] with the following primary antibodies: BrdU (5-bromodeoxyuridine; rat; AbD Serotec; 1∶40), Olig2 (oligodendrocyte transcription factor 2; Rabbit; Millipore; 1∶500), CC1 (*Adenomatous polyposis coli* (APC); Mouse, Calbiochem; 1∶200), GFAP (Glial Fibrillary Acidic Protein; Rabbit; Dako; 1∶200), and IBA1 (ionized calcium-binding adapter molecule 1; rabbit; Wako Pure Chemicals; 1∶1000). Blocking (1 h at room temperature) and subsequent antibody dilutions were performed in 10% normal goat serum/0.3% Triton X-100 in PBS. Antibodies were detected using appropriate species secondary antibodies conjugated to FITC, TRITC or AMCA (1∶200; Jackson ImmunoResearch, West Grove, PA). The secondary antibody incubation included Hoechst 33342 (1∶10000; Invitrogen, Carlsbad, CA) to visualize the nucleus of all cells. BrdU incorporation was detected as previously described [15].

For electron microscopy, mice were perfused as detailed above and brains were dissected and cut between Bregma −0.94 and −4.04. This region of brain between Bregma −0.94 and −4.04 was postfixed overnight at 4°C in 4% paraformaldehyde/2.5% glutaraldehyde in sodium cacodylate buffer pH 7.4. On the following day, the brain was cut sagittally, trimmed to expose the splenium of the caudal corpus callosum, washed in cacodylate buffer and processed for resin embedding. Sections were cut for Electron Microscopy and imaged on a Siemens Stereoskop Transmission Electron Microscope (Siemens, Munich, Germany) as previously described [16].

### Quantifications and statistical analysis

All cell counts and area analyses were performed blind to the experimental treatment. Immunopositive cells and area measures were quantified as previously described [15] and data are expressed as mean value/mm^2^ ±SEM. To quantify immunopositive cells, images were captured of coronal sections using a Zeiss Axioplan microscope (Zeiss, Thornwood, NY, USA) with a 20× objective. Images from three to six sections 50 µm apart were captured for each animal at locations between −0.46 mm and −1.22 mm Bregma. Images were oriented with the midline corpus callosum at the epicenter with the lateral borders determined by the width of the image. Electron micrograph quantification of myelinated axons was performed on four 3000× images per animal. The diameter measures of the g ratios (the ratio of axon diameter to the axon plus myelin sheath diameter) were calculated using Image J software for at least 200 fibers per animal.

All statistical tests were performed using GraphPad Prism (GraphPad Software). One-way ANOVA followed by Newman-Keuls multiple comparison post test was used for comparison between three to four groups unless otherwise stated. The Student's unpaired t test was used for comparison between two groups.

## Results

### Sequential delivery of BMP4 and Noggin during cuprizone challenge does not alter glial cell numbers or level of demyelination

**Figure 2:**
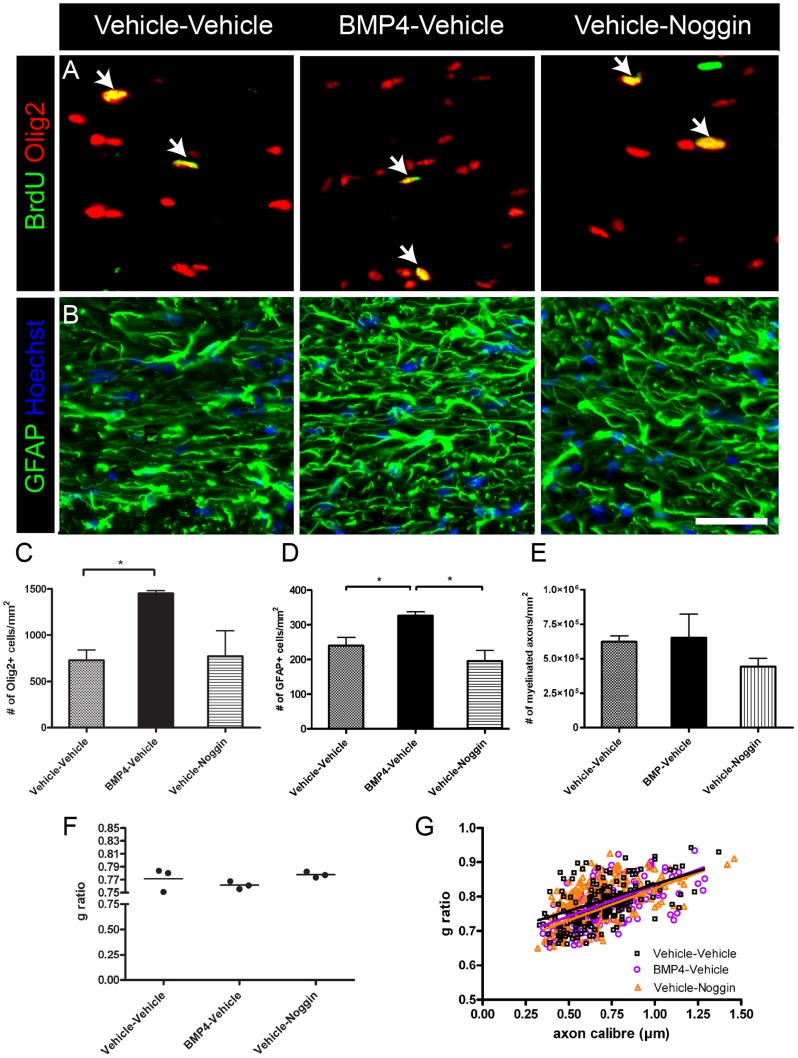
BMP4 infusion increases numbers of oligodendroglia and astrocytes during cuprizone challenge but does not influence the level of demyelination. (A–B) Immunostaining of BrdU-Olig2 (A) and GFAP (B) of the midline corpus callosum of the infused mice after 6-weeks cuprizone challenge. Arrows indicate double positive cells in A. Nuclei were counterstained with Hoechst in B. (C–D) Quantification of Olig2+ (C) and GFAP+ (D) in the midline corpus callosum of the infused mice after 6-weeks cuprizone challenge. (E–F) Quantification of the density (E) and average g ratio (F) of myelinated axons. (G) G ratios of individual axons as a function of axonal diameter in the corpus callosum of the infused mice after 6-weeks cuprizone challenge. n = 4–6 animals per group. *p<0.05,** *p*<0.01; Dunnett's multiple comparison post test in (C). Scale bar in B, 50 µm.

To determine the consequences of sequential delivery of BMP4 and Noggin during cuprizone challenge, mini-osmotic pumps were implanted after 4-weeks of cuprizone challenge to deliver either vehicle or BMP4 into the lateral ventricle for 7 days (Figure 1A). Next, the mini-osmotic pump delivering vehicle or BMP4 was replaced with a new pump delivering vehicle or Noggin for the final 7 days of a 6-week cuprizone challenge. In the experimental paradigm, mice received BrdU for the final three days of the first infusion. This timing of BrdU administration was selected in order to label cells that were generated during the initial infusion (i.e. BMP4 or vehicle). Initially, the baseline effects of BMP4 and Noggin single delivery during cuprizone challenge were assessed after six-weeks cuprizone challenge. The proliferation of oligodendroglial cells was examined, and it was found that BMP4-vehicle or vehicle-Noggin infusion did not significantly alter the density of BrdU+Olig2+ cells (BrdU+Olig2+: vehicle-vehicle 149±57.6/mm^2^; BMP4-vehicle 83.9±15.9/mm^2^; vehicle-Noggin 191±111/mm^2^; *p* = 0.60) or the percentage of Olig2+ cells that are BrdU+ (% Olig2+ cells that are BrdU+: vehicle-vehicle 28.0±14.1; BMP4-vehicle 5.83±1.24; vehicle-Noggin 25.1±9.36; *p* = 0.29). Furthermore, there was no significant difference in the density of BrdU+ cells in the infused mice (BrdU: vehicle-vehicle 454±190/mm^2^; BMP4-vehicle 157±30.5/mm^2^; vehicle-Noggin 316±123/mm^2^; *p* = 0.35). However, there was a significant increase in density of Olig2+ and GFAP+ cells in the corpus callosum of BMP4-vehicle infused mice compared to vehicle-vehicle (Figure 2A–D), while the density of GFAP+ cells was decreased in the vehicle-Noggin infused mice compared to BMP4-vehicle (Figure 2B,D). There was no change in the density of IBA1+ cells among the three groups of infused mice (IBA1+: vehicle-vehicle 596±281/mm^2^; BMP4-vehicle 327±78.0/mm^2^; vehicle-Noggin 736±115/mm^2^; *p* = 0.32).

For analysis of myelination, the ultrastructure of the caudal corpus callosum was examined by electron microscopy. After six-weeks of cuprizone challenge, there was no change in the density of myelinated axons or in myelin sheath thickness as represented by g ratio analysis (Figure 2E–G).

**Figure 3:**
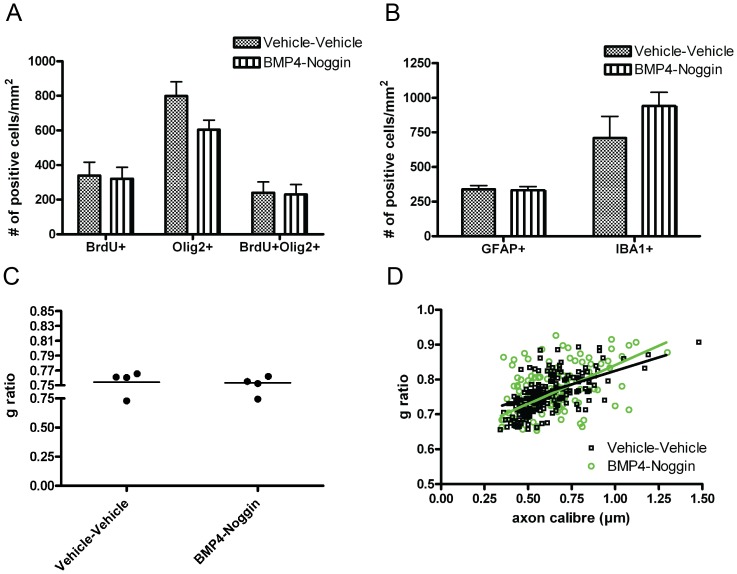
Sequential delivery of BMP4 and Noggin during cuprizone challenge does not alter numbers of glial cells or myelination. (A–B) Quantification of BrdU+, Olig2+, BrdU+Olig2+, IBA1+ and GFAP+ cells in the midline corpus callosum of the sequentially infused mice after 6-weeks cuprizone challenge. (C) Quantification average g ratio of myelinated axons. (D) G ratios of individual axons as a function of axonal diameter in the corpus callosum of the infused mice after 6-weeks cuprizone challenge. n = 4 animals per group.

After assessing the baseline effects of BMP4 and Noggin single delivery, we next investigated the effects of sequential delivery of BMP4 and Noggin during cuprizone challenge after 6-weeks of cuprizone challenge. Interestingly, sequential delivery of BMP4 and Noggin did not alter proliferation or numbers of oligodendroglial cells (Figure 3A). There was also no change in the density of GFAP+ cells in the corpus callosum of BMP4-Noggin infused mice compared to vehicle-vehicle or in the density of microglia cells (Figure 3B). Electron microscopy analysis also revealed no difference in the density of myelinated axons in the infused mice (myelinated axons: vehicle-vehicle 480721±82098/mm^2^; BMP4-Noggin 370864±148497/mm^2^; *p* = 0.54). Furthermore, g ratio analysis revealed no difference in myelin thickness (Figure 3C–D). Together, these data indicate that delivery of BMP4 alone during cuprizone challenge increases numbers of oligodendroglia and astrocytes which is consistent with previous work [8], however, sequential delivery of BMP4 and Noggin does not modulate glial cell numbers or influence the level of demyelination compared with vehicle infusion.

### BMP4 inhibits the influence of Noggin on mature oligodendrocyte regeneration and remyelination

**Figure 4:**
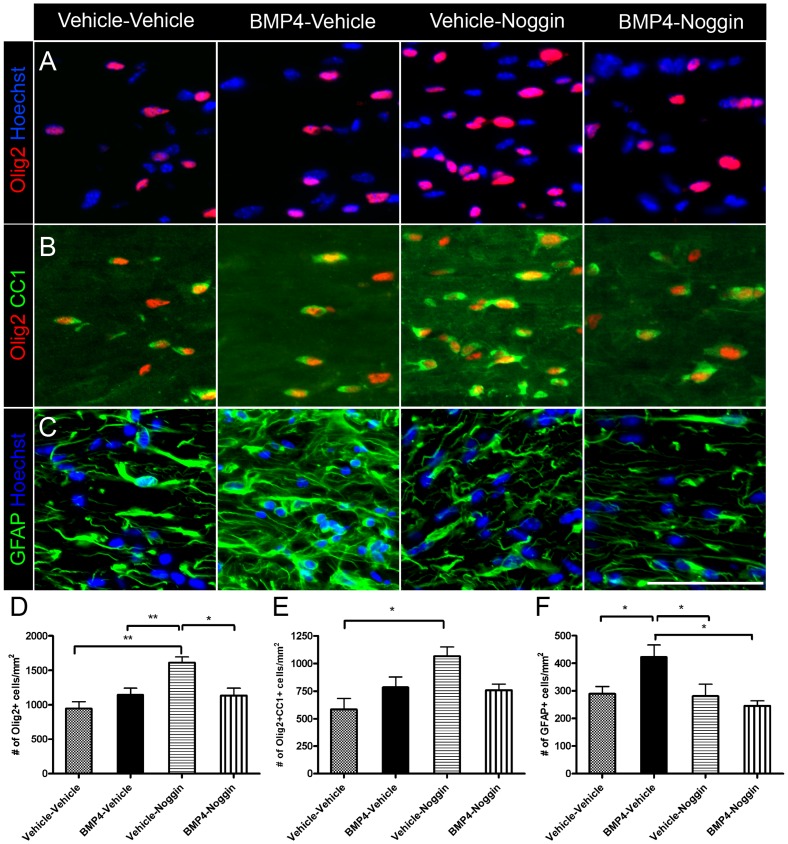
Sequential delivery of BMP4 and Noggin during cuprizone challenge does not alter numbers of oligodendrocytes and astrocytes following recovery. (A–C) Immunostaining of Olig2 (A), Olig2 and CC1 (B), and GFAP (C) in the midline corpus callosum of the sequentially infused mice following 1-week recovery. Nuclei were counterstained with Hoechst. (D–F) Quantification of Olig2+, Olig2+CC1+ and GFAP+ in the midline corpus callosum of the sequentially infused mice following 1-week recovery. n = 4–6 animals per group. *p<0.05,** *p*<0.01. Scale bars in C, 50 µm.

Given there was not an effect of sequential delivery of BMP4 and Noggin compared to vehicle during cuprizone challenge, we wanted to determine if sequential delivery of BMP4 and Noggin would instead influence oligodendrogliogenesis and remyelination during recovery from cuprizone challenge. For this experiment, mini-osmotic pumps were implanted after 4-weeks of cuprizone challenge to deliver either vehicle or BMP4 into the lateral ventricle for 7 days (Figure 1B). Next, the mini-osmotic pump delivering vehicle or BMP4 was replaced with a new pump delivering vehicle or Noggin for the final 7 days of a 6-week cuprizone challenge. Mice were allowed to recover for 1-week from six-week cuprizone challenge and received BrdU for the final three days of the first infusion (Figure 1B). Within the corpus callosum, there was no significant difference in the density of Olig2+ oligodendroglia (Figure 4A,D) or Olig2+CC1+ mature oligodendrocytes (Figure 4B,E) in the BMP4-Noggin infused mice compared to vehicle-vehicle infused mice. In addition, there was no significant difference in the density of BrdU+ cells, BrdU+Olig2+ and BrdU+CC1+ double positive cells in the corpus callosum of the infused mice (BrdU+: vehicle-vehicle 245±32.5/mm^2^; BMP4-vehicle 299±54.9/mm^2^; vehicle-Noggin 283±54.5/mm^2^; BMP4-Noggin 201±63.1/mm^2^; *p* = 0.57; BrdU+Olig2+: vehicle-vehicle 98.9±19.7/mm^2^; BMP4-vehicle 137±35.5/mm^2^; vehicle-Noggin 105±20.7/mm^2^; BMP4-Noggin 90.0±33.9/mm^2^; *p* = 0.69; BrdU+CC1+: vehicle-vehicle 43.0±9.29/mm^2^; BMP4-vehicle 36.3±12.3/mm^2^; vehicle-Noggin 73.3±12.1/mm^2^; BMP4-Noggin 35.4±16.8/mm^2^; *p* = 0.16). There was also no change in the density of GFAP+ cells in the corpus callosum of BMP4-Noggin infused mice compared to vehicle-vehicle (Figure 4C,F) or in the density of microglia cells (IBA1+: vehicle-vehicle 558±121/mm^2^; BMP4-vehicle 633±67.7/mm^2^; vehicle-Noggin 418±138/mm^2^; BMP4-Noggin 382±4.37/mm^2^; *p* = 0.35)

**Figure 5:**
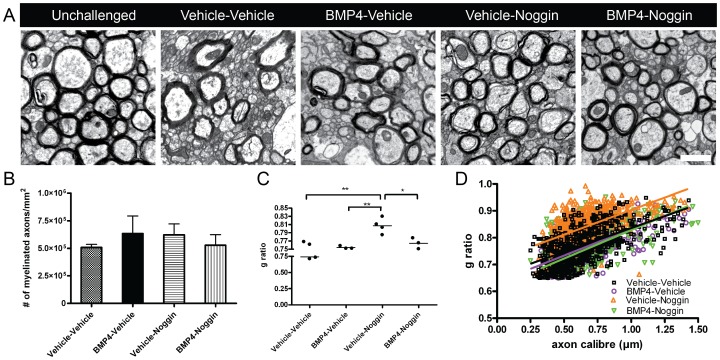
Sequential delivery of BMP4 and Noggin during cuprizone challenge does not enhance remyelination following recovery. (A) Representative electron micrographs in the caudal corpus callosum of unchallenged and sequentially infused mice after 1-week recovery. (B–C) Quantification of the density (B) and average g ratio (C) of myelinated axons. (D) G ratios of individual axons as a function of axonal diameter in the corpus callosum of the infused mice after 1-week recovery. n = 3–4 animals per group. *p<0.05. **p<0.01 Scale bar, 1 µm.

However, there were significant differences among the group of infused mice in terms of numbers of oligodendroglial and astroglial cells. Vehicle-Noggin infusion increased the density of Olig2-positive cells and Olig2-CC1 double-positive cells in the corpus callosum compared to vehicle-vehicle, while the density of Olig2-positive cells was reduced in the BMP4-Noggin infused mice compared to vehicle-Noggin (Figure 4A,B,D,E). BMP4-vehicle infusion increased the density of GFAP+ cells compared to vehicle-vehicle, whereas BMP4-Noggin infusion decreased the density of GFAP+ cells compared to BMP4-vehicle (Figure 4C,F). Remyelination was examined by electron microscopy and no change was found in the density of myelinated axons in the sequentially infused mice (Figure 5B). In addition, there was no significant difference in the average g ratio of the myelinated axons in the corpus callosum of the BMP4-Noggin infused mice compared to vehicle-vehicle infused mice (Figure 5A, C). However, g ratio analysis revealed higher g-ratios of myelinated axons in the corpus callosum of the vehicle-Noggin infused mice as opposed to the other groups of mice, suggesting an increase in thinly myelinated axons (Figure 5A, C, D).

Taken together, these data indicate that Noggin delivery alone increases the density of mature oligodendrocytes, while BMP4 delivery alone increases the density of astrocytes, which are decreased by sequential delivery of BMP4 and Noggin. The results also suggest that sequential delivery of BMP4 and Noggin does not further enhance mature oligodendrocyte regeneration and remyelination above what occurs with Noggin delivery alone.

### Sequential delivery of BMP4-IGF-1 during cuprizone challenge increases numbers of mature oligodendrocytes but does not enhance remyelination

**Figure 6:**
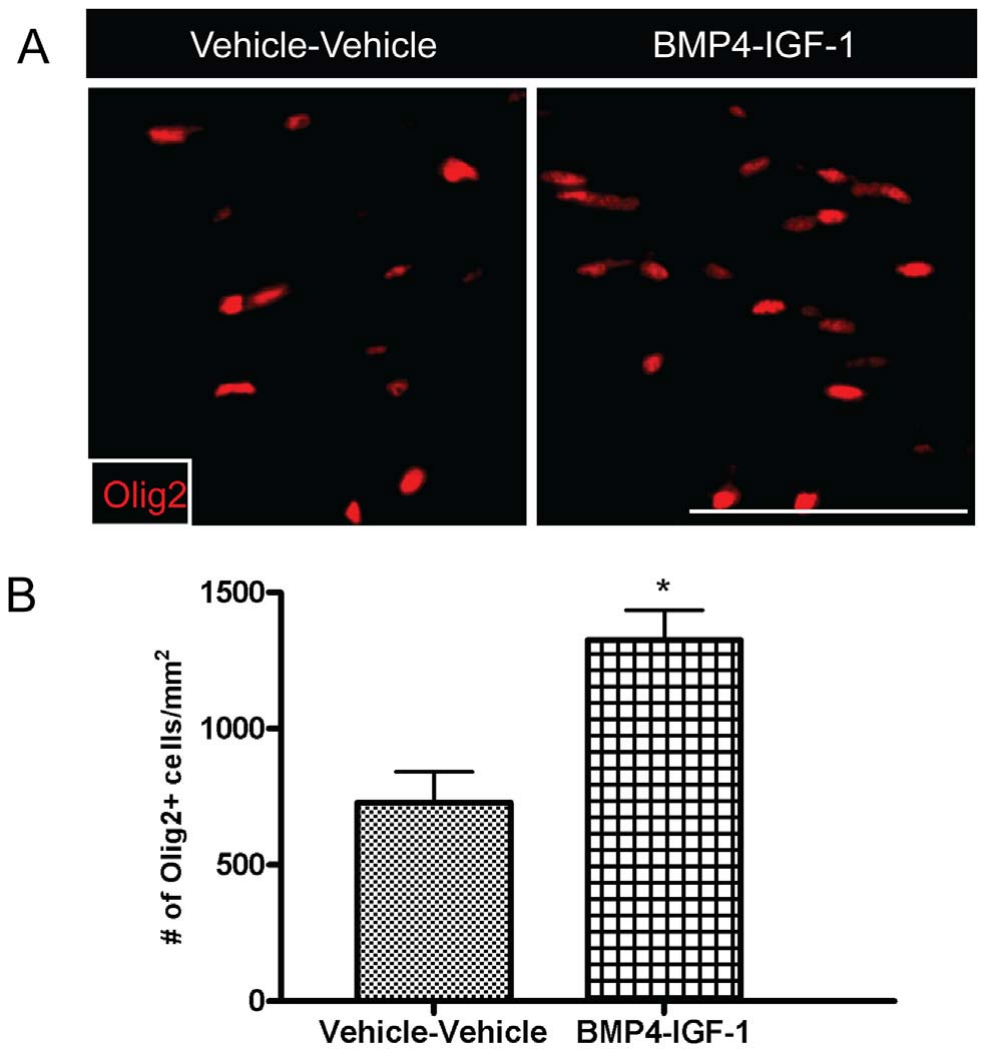
Sequential delivery of BMP4 and IGF-1 during cuprizone challenge increases numbers of oligodendroglia in the corpus callosum. (A) Immunostaining of Olig2 in the corpus callosum of the sequentially infused mice after 6-weeks of cuprizone challenge. (B) Quantification of the density of Olig2+cells in the corpus callosum of the sequentially infused mice after 6-weeks of cuprizone challenge. n = 3 animals per group. *p<0.05. Scale bar, 50 µm.

There is *in vitro* and *in vivo* evidence that insulin-like growth factor-1 (IGF-1) promotes the survival of oligodendrocytes and myelination [9], [12]. Previous work has shown that BMP4 delivery during cuprizone challenge increased numbers of OPCs, however, there was evidence for increased apoptosis and decreased oligodendroglia in the corpus callosum in BMP4-infused mice following recovery [8]. Therefore, we hypothesized that delivery of IGF-1 following BMP4 during cuprizone challenge could enhance myelin repair by increasing the survival of the newly generated OPCs. For these experiments, mice were sequentially infused with BMP4 and IGF-1 during cuprizone challenge and assessments were made at six-weeks of cuprizone and 1-week recovery (Figure 1). Similar to the results for the BMP4-vehicle infusion in Figure 2C, there was a significant increase in the density of Olig2+ oligodendroglia in the corpus callosum of BMP4-IGF-1 infused mice compared to vehicle-vehicle after six-weeks of cuprizone challenge (Figure 6). However, there was no difference in the density of BrdU+ and BrdU+Olig2+ cells in the infused mice (BrdU+: vehicle-vehicle 454±190/mm^2^; BMP4-IGF-1 336±132/mm^2^; *p* = 0.64; BrdU+Olig2+: vehicle-vehicle 149±57.6/mm^2^; BMP4-IGF-1 101±13.7/mm^2^; *p* = 0.46). Similarly, BMP4-IGF-1 infusion did not alter the density of GFAP+ or IBA1+ cells (GFAP+: vehicle-vehicle 240±23.3/mm^2^; BMP4-IGF-1 220±27.0/mm^2^; *p* = 0.60; IBA1+: vehicle-vehicle 596±281/mm^2^; BMP4-IGF-1 383±13.5/mm^2^; *p* = 0.52). There was also no difference in the level of myelination in the infused mice as assessed by electron microscopy (myelinated axons: vehicle-vehicle 624359±41760.0/mm^2^; BMP4-IGF-1 811604±208273/mm^2^; *p* = 0.43; g ratio: vehicle-vehicle 0.772±0.010; BMP4-IGF-1 0.760±0.014; *p* = 0.53).

**Figure 7:**
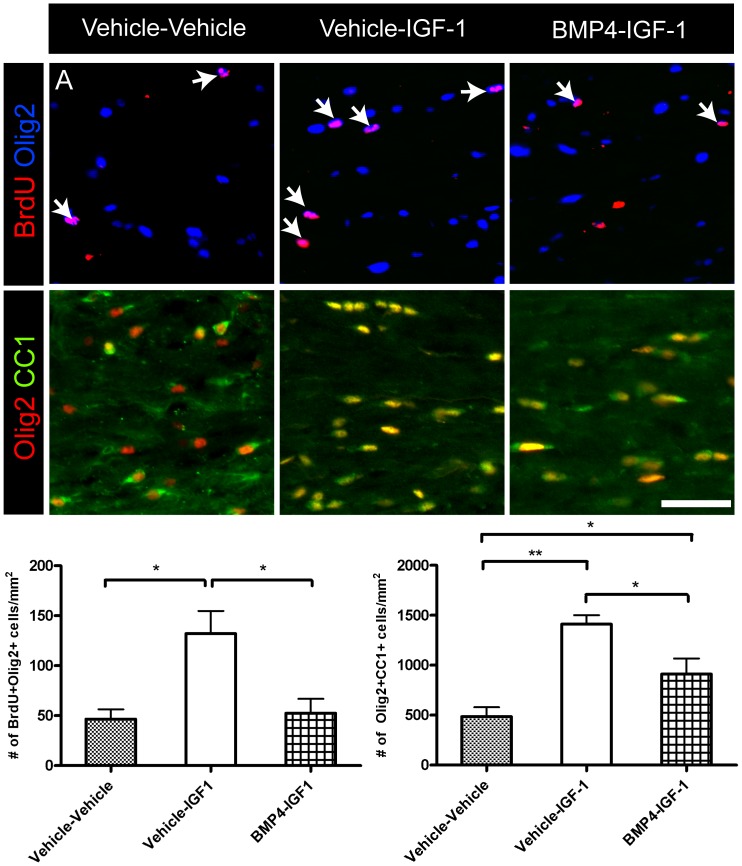
Sequential delivery of BMP4 and IGF-1 during cuprizone challenge increases numbers of mature oligodendrocytes following recovery. (A–B) Immunostaining of BrdU-Olig2 (A) and Olig2-CC1 (B) in the corpus callosum of the sequentially infused mice after 1-week recovery. (C,D) Quantification of the density of BrdU+Olig2+ (C) and Olig2+CC1+ (D). n = 3–4 animals per group. *p<0.05. Scale bar, 50 µm.

**Figure 8:**
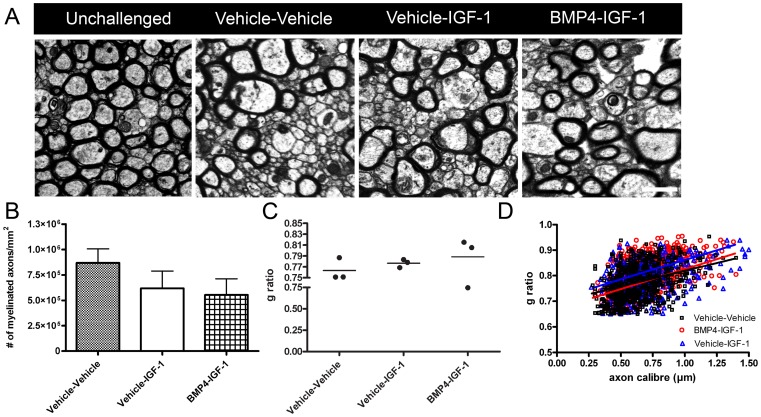
Sequential delivery of BMP4 and IGF-1 during cuprizone challenge does not enhance remyelination. (A) Representative electron micrographs in the caudal corpus callosum of unchallenged and sequentially infused mice after 1-week recovery. (B–C) Quantification of the density (B) and average g ratio (C) of myelinated axons. (D) G ratios of individual axons as a function of axonal diameter in the corpus callosum of the infused mice after 1-week recovery. n = 3 animals per group Scale bar, 1 µm.

Following 1-week recovery from cuprizone challenge, Olig2+CC1+ cells were significantly increased in the BMP4-IGF-1 infused mice compared to vehicle-vehicle (Figure 7B,D). However, there was no difference in the density of BrdU+Olig2+ cells in the BMP4-IGF-1 infused mice compared to vehicle-vehicle (Figure 7A,C). Vehicle-IGF-1 infusion increased numbers of oligodendrocytes compared to vehicle-vehicle, whereas BMP4-IGF-1 decreased numbers of oligodendrocytes compared to vehicle-IGF-1 (Figure 7). When numbers of astrocytes were examined at 1-week recovery, GFAP+ cells were significantly decreased in the BMP4-IGF-1 and vehicle-IGF-1 infused mice compared to vehicle-vehicle (GFAP+: vehicle-vehicle 551±70.7/mm^2^; vehicle-IGF-1 296±48.6/mm^2^; BMP4-IGF-1 264±14.3/mm^2^; *p*<0.05) There was no difference in the density of IBA1+ cells in the three groups of infused mice at 1-week recovery (IBA1+: vehicle-vehicle 385±88.8/mm^2^; vehicle-IGF-1 520±102/mm^2^; BMP4-IGF-1 499±112/mm^2^; *p* = 0.63). In addition, the ultrastructure of the caudal corpus callosum was examined in the sequentially infused mice following 1-week recovery (Figure 8A). There was no change in the density or average g ratio of myelinated axons or g ratios in relation to axon caliber among the three groups of mice (Figure 8B–D).

These results suggest that delivery of IGF-1 during cuprizone challenge increases oligodendrocyte numbers and decreases astrocyte numbers following recovery from cuprizone challenge, however, remyelination is not enhanced. Furthermore, sequential delivery of BMP4 and IGF-1 does not increase mature oligodendrocyte regeneration above what occurs with IGF-1 infusion alone.

## Discussion

This study has described the consequences of sequential delivery of growth factors during cuprizone-induced demyelination. Sequential delivery of BMP4 and Noggin during cuprizone challenge did not alter numbers of oligodendrocytes or astrocytes following recovery. Furthermore, evidence from electron microscopy suggested there was not enhanced remyelination in the BMP4-Noggin infused mice. In contrast, sequential delivery of BMP4 and IGF-1 during cuprizone challenge increased the number of mature oligodendrocytes and decreased numbers of astrocytes following recovery. However, further investigation by electron microscopy, suggested that BMP4-IGF-1 delivery did not alter remyelination.

Several recent studies have shown that sequential delivery of growth factors is a promising repair strategy. For instance, sequential delivery of basic fibroblast growth factor (bFGF) and sonic hedgehog enhanced bone regeneration *in vivo* [17]. Malignant tumours were eradicated in mice following interleukin-12 and -27 sequential gene therapy [18]. Furthermore, Ruvinov *et al* [19] showed that sequential delivery of IGF-1 and hepatocyte growth factor promoted myocardial repair with increased angiogenesis occurring at the infarct. Similarly, sequential delivery bFGF and platelet-derived growth factor resulted in functional angiogenesis *in vivo* [20]. This is the first study to explore the effects of sequential growth factor delivery on myelin repair *in vivo*.

There is evidence to suggest that Noggin potentiates the differentiation of OPCs into remyelinating oligodendrocytes *in vivo*. Noggin infusion during cuprizone challenge increased the number of mature oligodendrocytes and remyelinated axons following recovery [8]. Given that OPC differentiation is a major step in the remyelination process [3], Noggin could be a potential candidate to promote the differentiation of the OPCs generated by BMP4 which would be advantageous for repair. Therefore, it is plausible that sequential delivery of BMP4 and Noggin would result in enhanced mature oligodendrocyte regeneration and myelin repair. However, sequential delivery of BMP4 and Noggin during cuprizone challenge did not increase numbers of oligodendroglia following recovery from cuprizone challenge. BrdU was administered during the final three days of BMP4 infusion during weeks 4–5 cuprizone and, at 1-week recovery, there was no difference in the numbers of BrdU+ cells that incorporated Olig2 and CC1 in BMP4-Noggin infused mice compared to vehicle-vehicle infused mice. This would indicate that Noggin did not promote the survival or differentiation of the OPCs generated by BMP4 infusion. However, there were increased numbers of mature oligodendrocytes in the corpus callosum of vehicle-Noggin infused mice compared to vehicle-vehicle infused mice. These data are consistent with a previous report [8] and suggests that Noggin is enhancing the differentiation of resident OPCs in the corpus callosum. Thus, the absence of a positive effect in the number of mature oligodendrocytes at 1-week recovery with sequential delivery of BMP4 and Noggin compared to vehicle-vehicle could be attributed to Noggin acting on cells resident in the corpus callosum rather than on the additional OPCs generated by increased proliferation in response to BMP4 infusion.

A major challenge in sequential delivery of growth factor antagonists is regulation of the exogenous and endogenous levels of signalling. In this study, BMP signalling was initially increased with BMP4 infusion and then decreased with Noggin infusion. Interestingly, when assessed during recovery, oligodendrogliogenesis was decreased in the BMP4-Noggin infused mice compared to vehicle Noggin mice, suggesting that Noggin following BMP4 infusion could not inhibit the high level of exogenous BMP4, which may be inhibitory for oligodendrogliogenesis. While the level of BMP signalling was not assessed in this study, a high level of BMP signalling could explain the lack of enhanced repair following sequential delivery of BMP4 and Noggin. Conversely, astrocyte numbers were decreased in the corpus callosum of the BMP4-Noggin infused mice compared to BMP4-vehicle infused mice. This suggests that Noggin following BMP4 infusion can decrease the BMP4-induced increase in astrocytes. In animal models of demyelination [15] and intraventricular hemorrhage [21], where levels of endogenous BMP signalling are increased, Noggin delivery increases oligodendrocyte numbers and decreases astrocyte numbers. Therefore, while exogenous Noggin inhibits the effects of endogenous BMP signalling on numbers of oligodendrocytes and astrocytes, Noggin could have limited capacity to inhibit the effects of exogenous BMP4 on oligodendrocytes.

An important finding in this study was the decrease in mature oligodendrocytes in the BMP4-IGF-1 infused mice compared to the vehicle-IGF-1 infused mice. There is *in vitro* evidence that IGF-1 regulates BMP activity. For example, Noggin gene expression was upregulated following IGF-1 induced oligodendrocyte differentiation of adult neural precursor cell cultures [22]. Wahdan-Alaswad *et al* [23] reported that IGF-1 suppressed BMP4-induced apoptosis, BMP signalling activation and expression of inhibitors of differentiation/DNA binding (Id) proteins in prostrate epithelial cells through activation of mammalian target of rapamycin signalling. The Ids are downstream BMP target genes and mediate the inhibitory effects of BMPs on oligodendrocyte differentiaton [24]. Therefore, a potential molecular mechanism to explain IGF-1 mediated effects on oligodendrocyte differentiation may involve repression of the Id proteins. In this study, IGF-1 did not promote the survival of the OPCs generated by BMP4, given that at 6-weeks cuprizone and 1-week recovery, there was no difference in the number of BrdU+Olig2+ cells in BMP4-IGF-1 and vehicle-vehicle infused mice. Furthermore, BrdU+Olig2+ and Olig2+CC1+ cells were decreased in the corpus callosum of the BMP4-IGF1 infused mice compared to vehicle-IGF-1 infused mice at 1-week recovery, suggesting that BMP4 is blocking the capacity for the IGF-1 induced increase in oligodendrogliogenesis. Thus, it appears that the OPCs generated by BMP4 infusion may be prone to cell death and not responsive to IGF-1 meditated survival. In addition, the IGF binding proteins (IGFBP) can regulate IGF-1 activity by inhibiting its interaction with the type I IGF receptor [25], and reports have indicated that IGFBP-1 inhibits the effects of IGF-1 on oligodendrocyte survival and myelination *in vitro* and *in vivo* [12], [26]. Kühl *et al* [26] showed that exogenous IGFBP-1 reduced the IGF-1 stimulated survival and differentiation of OPCs *in vitro* [26]. Furthermore, transgenic overexpression of IGFBP-1 decreased myelin gene expression and numbers of myelinated axons in the brain [12]. There is also evidence to suggest that IGFBPs regulate BMP-induced activity in glomerular podocytes and mesenchymal stem cells [27], [28]. Given that IGF-1 following BMP4 infusion did not enhance OPC survival, this could suggest that BMP signalling is inducing an IGF-1 dependent effect of IGFBP-1, which would repress the survival activity of IGF-1 on oligodendrocytes. It can also be concluded from this study that IGF-1 infusion alone promotes mature oligodendrocyte regeneration following recovery from central demyelination.

Several studies have reported that IGF-1 promotes CNS myelination *in vivo* [11], [12], however, the role of IGF-1 in remyelination is less clear, particularly when it is exogenously delivered. In EAE animals, systemic delivery of IGF-1 during the acute phase reduced the area of demyelinated lesions in the spinal cord [29] but failed to have any effect on remyelination as revealed by electron microscopy when delivered during the chronic phase [30]. Furthermore, adeno-associated viral delivery of IGF-1 in the spinal cord of EAE animals actually worsened clinical symptoms [31]. O'Leary *et al* [32] reported no effect on oligodendrocyte remyelination following delivery of IGF-expressing adenovirus directly into lysolecithin-induced demyelinated lesions of aged animals. Moreover, administration of IGF-1 to a small cohort of multiple sclerosis patients did not alter clinical disease activity [33]. In this study, intraventricular infusion of IGF-1 during cuprizone-induced demyelination did not increase thinly myelinated, presumably remyelinated, axons despite there being an increase in mature oligodendrocytes in these animals.

The aim of this study was to determine whether sequential delivery of BMP4 and Noggin or BMP4 and IGF-1 would further enhance myelin repair. While sequential delivery of BMP4 followed by either Noggin or IGF-1 did not further enhance myelin repair as hypothesised, the experimental findings also highlighted the difference in the effects of Noggin and IGF-1 on remyelination. Both growth factors increased the production of mature oligodendrocytes *in vivo* in the context of demyelination: however, whereas Noggin alone promoted an increase in the number of thinly myelinated axons IGF-1 alone did not do so. This is consistent with the idea that Noggin is increasing the differentiation of OPCs into remyelinating oligodendrocytes, while IGF-1 is increasing the survival of mature oligodendrocytes during the insult but that it may not actively contribute to the remyelination process, at least under these circumstances. In support of this, there is evidence to suggest that surviving mature oligodendrocytes fail to remyelinate lesions [34]. Keirstead et al [34] irradiated the spinal cord prior to focal demyelination and showed that postmitotic oligodendrocytes survive within the demyelinated lesion and do not contribute to remyelination. Thus, therapeutic approaches targeting myelin repair will have to consider not only the potentiation of oligodendrocyte survival but also their capacity to remyelinate.

In summary, this study has revealed important insights into the complexity of sequential growth factor delivery systems and their ability to influence repair within the context of demyelination. The results presented here indicate that sequential delivery of BMP4 and Noggin or IGF-1 does not further enhance myelin repair above what occurs with delivery of Noggin alone. Further work is required to elucidate the molecular mechanisms that occur in OPCs in response to increased BMP signalling which could lead to the identification of a growth factor that potentiates the survival and differentiation of these cells to further enhance myelin repair.
